# The Impact of Narrative Strategy on Promoting HPV Vaccination among College Students in Korea: The Role of Anticipated Regret

**DOI:** 10.3390/vaccines8020176

**Published:** 2020-04-10

**Authors:** Jarim Kim

**Affiliations:** Department of Communication, Yonsei University, 50 Yonsei-ro, Seodaemun-gu, Seoul 03722, Korea; jarimkim@yonsei.ac.kr

**Keywords:** narrative, communication strategy, anticipated regret, health communication, HPV, vaccine

## Abstract

Human papillomavirus (HPV) vaccine hesitancy contributes to unsatisfactory vaccination coverage in Korea despite its high efficacy in preventing various diseases including cervical cancer. To enhance HPV vaccine uptake, effective communication with the public is key. To develop effective health promotion messages, this study examined the effects of message format on attitudes and intentions toward HPV vaccination, specifically focusing on anticipated action and inaction regrets. It employed a randomized experimental message design format (narrative versus didactic messages). A total of 222 Korean undergraduate students who had not received the HPV shot participated in the experiment. The results showed that didactic messages produce greater anticipated inaction regret, which further influences HPV vaccination attitudes and behaviors. Anticipated regret could potentially explain mixed narrative effects across health behaviors as described in existing literature.

## 1. Introduction

Human papillomavirus (HPV) is one of the most pervasive sexually transmitted diseases and can develop into cervical and anal cancers [1]. In 2012, there were 266,000 worldwide terminal cases of cervical cancer, the fourth most prevalent female cancer [2]. The HPV vaccine is acknowledged to prevent approximately 70% of cervical cancer cases as well as other diseases including anal cancer and genital warts [3,4]. Despite its high efficacy and safety, the HPV vaccine remains unacceptable to large swaths of the public; this unacceptability has various causes including concerns about the vaccine’s side effects, questions about its necessity, and parents’ fears about causing their children to instigate sexual activity [5,6,7]. More recently, misinformation surrounding vaccine safety such as the controversy linking autism and vaccination has increased exponentially [8,9]. In part, unsatisfactory vaccine coverage may be due to inefficacy in HPV vaccine communication. Effective communication may increase acceptance of the HPV vaccine, ultimately helping to relieve the public health burden of HPV and prevent life-threatening cancer.

To this end, the present study focused on the relative effectiveness of narrative message formats, which have been acknowledged as “a promising set of tools for motivating and supporting health-behavior change” [10] (p. 789). Specifically, this study explored the mediating role of anticipated regret, a key determinant of health decision-making [11]. Thus far, researchers have emphasized the advantages of narrative effects while paying little attention to the processes underlying their disadvantages. By revealing the role of anticipated regret, this study aimed to enhance our understanding of narrative effects—regarding which studies have produced mixed findings [12]—and ultimately contribute to the creation of effective health communication messages.

The present study focused on Asian young adults because HPV vaccination coverage is much lower in Asia (1.1%) compared to other continents (e.g., 35.6% in North America) [13]. Moreover, young adults are at high risk of HPV infection; young adults between 18 and 26 years old account for 75% of new HPV incidences [14]. Despite the low coverage and high infection rates among Asian young adults, a dearth of research has been conducted with this population.

### 1.1. Narrative Message Format

Narrative, defined as “a representation of connected events and characters that has an identifiable structure, is bounded in space and time, and contains implicit or explicit messages about the topic being addressed” [15] (p. 222), is a term that includes anecdotes, testimonials, stories, and exemplars. It is often contrasted with non-narratives such as expository messages, didactic arguments, or statistical information [10,15]. For example, Janssen, Van Osch, De Vries, and Lechner [16] compared the relative effectiveness of didactic (i.e., fact-based risk information) and narrative (i.e., personal testimonial) messages and found that narrative messages are more effective in increasing risk perceptions. In health promotion contexts, scholars have examined narratives because this specific message format is expected to effectively engage individuals, adopt natural forms of human communication, and consequently make particular messages more “contextual and meaningful” [17] (p. 495). By transporting individuals into an “integrative melding of attention, imagery, and feelings” [18] (p. 164), narratives make message recipients focus on the events of the messages and evoke emotional reactions rather than developing counterarguments [19,20]. A recent meta-analysis supported this argument, showing that narratives are generally more effective than non-narratives in promoting health behaviors [21]. However, the study revealed that the effect was not significant when the messages were provided in print materials (vs. audio and video materials) [21]. Another systematic review [12] argued that it is premature to conclude that narratives are more effective than non-narratives.

In the context of vaccination, narrative effects are more obscure and related research has therefore produced even more mixed results than in other topic areas. For example, Hopfer’s longitudinal study [1] featuring female college students reported somewhat mixed results. The author exposed participants to three video messages acted out by peers, medical experts, or both and examined vaccine uptake after two months. The results showed that the narrative significantly increased vaccination rates only when the video featured both peers and experts and its effects were mediated by self-efficacy and intentions toward vaccination.

In contrast, Kim and Nan presented opposing results in an experimental study [22]. They exposed college students to a short message promoting HPV vaccination and found that participants in the non-narrative condition perceived HPV as more severe and getting the HPV vaccine as more beneficial than those in the narrative condition. This improved their attitudes toward the vaccine and increased their vaccination intentions. In a similar text message-based experimental study, Nan, Futerfas, and Ma found that participants in the non-narrative condition perceived similar levels of risk as participants in the first-person narrative condition where the patient used the pronoun “I” to relate her experience [23]. Participants in these two conditions, however, showed higher levels of risk perceptions than those in the third-person narrative condition, where a female name replaced the I used in the first-person condition. 

Nan et al.’s experiment with college students also produced null effects, showing no significant differences between narrative and non-narrative texts promoting the HPV vaccine [24]. These null effects remained when the narrative and non-narrative messages were presented via audio [23]. Nan et al. [24] pointed out that the “degree of narrativity” (p. 306) among various stimuli used in prior studies (e.g., long story, short texts) might explain such inconsistent results. The limited nature of previous research in this regard highlights the need further empirical investigation. Replicating Kim and Nan’s prior research [22], this study proposed the following hypotheses (Hs).

**Hypothesis 1** **(H1).** *A didactic message produces more positive attitudes toward HPV vaccination than a narrative message.*

**Hypothesis 2** **(H2).** *A didactic message produces greater intentions toward HPV vaccination than a narrative message.*

### 1.2. Anticipated Regret

Regret, “an aversive cognitive emotion” [11] (p. 1264), is experienced when we recognize that it would have been better if we had made alternative decisions in the past [25]. Having both cognitive and affective components, anticipated regret refers to “the expected post-behavioral affective consequences” [26] (p. 551) of either engaging in a behavior (action regrets; e.g., receiving a vaccine) or not engaging in a behavior (inaction regrets; e.g., not receiving a vaccine) that helps one achieve a goal (e.g., preventing cancer) [11]. Anticipated regret has been acknowledged as a critical determinant in various health contexts—including safe sex [26,27], exercise [28], smoking [29], and vaccination [30,31]—and as a predictor of health decisions above and beyond other factors [11,26].

When making health-related decisions, individuals forgo situations that would have resulted from their alternative decisions and feel responsible for the choices that lead to negative outcomes [11]. Anticipated regret is associated with getting or losing opportunities to perform given behaviors [32,33]. Regret management theory [25] explains that individuals attempt to avoid *self-blame* by minimizing anticipated regrets. In the context of HPV vaccination, two types of anticipated regrets can be evoked. Anticipated action regrets are evoked when one imagines performing a given behavior (e.g., imagining vaccine-related side effects), while anticipated inaction regrets occur when one imagines *not* performing a given behavior (e.g., not receiving the HPV vaccine and getting cervical cancer as a consequence). As individuals attempt to reduce self-blame after engaging in or avoiding certain behaviors, non-narrative messages—which rely on the credibility of medical authorities—are expected to be more effective than narrative messages—which are commonly presented by laypersons—in increasing anticipated inaction regrets. Anticipated action regrets, on the other hand, are expected to be more effective when the messages are presented by laypersons since individuals may think of possible procedural risks such as side effects [34]. Thus, the following hypotheses were proposed:

**Hypothesis 3** **(H3).** *A didactic message produces greater anticipated inaction regrets for not getting the HPV vaccine than a narrative message.*

**Hypothesis 4** **(H4).** *A didactic message produces less anticipated action regrets for getting the HPV vaccine than a narrative message.*

It is further postulated that non-narratives have indirect effects on vaccinating attitudes and intentions via anticipated regrets since anticipated regrets are a critical predictor of HPV vaccination behavior [31,35]. For example, Cox, Sturm, and Cox found that when mothers presented with graphical representations of risk information were asked about anticipated regrets, their intentions to get their daughters vaccinated against HPV increased [36]. Prior studies (e.g., [37]) have focused on cognitive mediators such as counter-arguing. Anticipated regret, both affective and cognitive, is expected to mediate such relationships.

**Hypothesis 5** **(H5).** *Message format has an indirect effect on attitudes through anticipated inaction regrets.*

**Hypothesis 6** **(H6).** *Message format has an indirect effect on intentions through anticipated inaction regrets.*

**Hypothesis 7** **(H7).** *Message format has an indirect effect on attitudes through anticipated action regrets.*

**Hypothesis 8** **(H8).** *Message format has an indirect effect on intentions through anticipated action regrets.*

## 2. Materials and Methods 

### 2.1. Sample and Design

To determine sample size, G*Power [38] was used with the alpha level set at 0.05, power at 0.80, and small-to-medium effect size (f = 0.20). Prior meta-analyses on narrative message effects have reported small-to-medium effect sizes [21,39]. The analysis required at least 199 participants to detect an effect.

For this study, 222 undergraduate students who had not received any HPV shots were recruited from communication classes at a major university in Seoul, Korea. The sensitivity power analysis for an ANCOVA based on G*Power [38] indicated that this sample size would allow the researcher to detect a small-to-medium effect size (f = 0.189). The sample’s mean (*M*) age was 21.22 years (standard deviation; *SD* = 2.28) and 53.2% of participants were females. Participation was voluntary and no rewards (e.g., extra credits) were provided. The HPV vaccine has been approved for both males and females aged 9–26 [40], and thus, both sexes were invited to take part in this study.

An experiment was conducted using a paper-and-pencil survey in classrooms. At the end of various classes, the researcher briefly introduced the study to the students and stated that their participation was voluntary. Those who were unwilling to take part in the survey left the classroom and those who were willing to take part remained and received the survey. Study participants first answered some questions regarding HPV and the HPV vaccine. They were then assigned to one of two experimental conditions: 112 participants were assigned to the didactic condition while 124 were assigned to the narrative condition. After reading the stimuli, they answered the survey questions regarding their anticipated regret, attitudes, intentions, and demographic information. A total of 15 surveys were excluded because they were either incomplete or marked repeatedly with the same number from the scale. Therefore, the final sample size was 221 (105 in the didactic condition and 116 in the narrative condition). The researcher obtained informed consent from the participants and endeavoured to comply with ethical standards for treating participants and conducting research, although the institution to which the researcher belonged while collecting data did not require such processes.

### 2.2. Stimuli

Two experimental stimuli were adapted from a prior study of narrative health messaging [22]. Each message, composed of three short paragraphs, was presented as an unformatted text provided by the Korean Association of Obstetrics and Gynecology, which actually conducts campaigns promoting HPV vaccination in Korea. The first and second paragraphs presented basic information regarding HPV and the vaccine and were identical for both conditions. Since the study was conducted in Korea, an additional statement “According to a survey with Korean female college students, about 15.2% were found to be infected by HPV” was added at the end of the first paragraph.

The message format (narrative vs. didactic) was manipulated in the last paragraph of each message. The didactic message stated: “The HPV vaccine works effectively to protect your body. Imagine the huge sense of relief you will feel after you have received the HPV vaccine!” Meanwhile, the narrative message stated: “‘I know the HPV vaccine works effectively to protect my body,’ said Yena, a University of X student who recently got vaccinated against HPV, ‘after I got the HPV vaccine, I felt a huge sense of relief!’” Appendix A presents the stimuli in their entirety.

### 2.3. Measures

#### 2.3.1. Anticipated Regret 

Anticipated regret, the extent to which one anticipates negative emotional consequences, was measured twice in relation to either acting (i.e., anticipated action regret) or not acting (i.e., anticipated inaction regret). Three items adapted from prior research [26,41] were used to measure these anticipated action/inaction regrets. Participants were asked to rate their responses (on three 7-point scales including “not regretful-regretful,” “not sorry-sorry,” and “not worried-worried”) to statements concerning inaction regret (e.g., “If I am offered the chance to take the HPV vaccine in the next few years and I do not take part, I will feel, …”) and action regret (e.g., “If I am offered the chance to take the HPV vaccine in the next few years and I take part, I will feel, …”). The three inaction regret items were averaged to form an index of anticipated inaction regret and the three action regret items were averaged to form an index of anticipated action regret. Higher scores indicated higher levels of regret. Table 1 presents descriptive statistics for key measures.

#### 2.3.2. Attitudes and Intentions 

Attitudes toward the HPV vaccination were measured using three items. Participants were asked to rate their responses on three 7-point semantic differential scales (“very foolish-very wise,” “very bad-very good,” and “very harmful-very beneficial”) to the statement “Getting vaccinated against the human papillomavirus (HPV) is, …” 

Intentions were measured using two scenarios: (1) the vaccine is offered free of charge and (2) the vaccine costs approximately $440. Intentions toward the HPV vaccination when offered for free were measured using three items. Participants were asked to envision themselves in a situation in which the HPV vaccine was offered for free and then to rate their responses on a 1–7 scale (e.g., 1 = very unlikely; 7 = very likely) to the following three questions: “How likely would you be to get the HPV vaccine in the future?”, “How likely would you be to get the HPV vaccine sometime soon?”, and “If you were faced with the decision of whether to get the HPV vaccine today, how likely is it that you would choose to get the vaccine?”. Intentions toward the vaccine with cost were measured with the same three items, after participants were asked to envision themselves in a situation in which they needed to pay $440 (i.e., approximately 0.5 million Korean Won) to get the vaccine. These measures were adapted from past studies [24,42,43].

## 3. Results

### 3.1. Manipulation Checks

For manipulation checks of the didactic and narrative message formats, an ANOVA was conducted. The results showed that the participants perceived the didactic message as more explanatory (didactic condition *M* = 4.86, *SD* = 1.37; narrative condition *M* = 4.29, *SD* = 1.46; $F_{1,220}$ *p* = 0.003) and narrative message as more focused on experience-based description (didactic condition *M* = 4.09, *SD* = 1.63; narrative condition *M* = 4.90, *SD* = 1.57; $F_{1,220}$ = 14.25, *p* = 0.000).

### 3.2. Main Test Results

To examine H1-H4, a series of ANCOVAs with the stimulus of narrative and didactic messages as independent variables and attitudes, intentions, and anticipated regret as dependent variables were conducted. Age and gender were included as control variables throughout the analyses. The results revealed a significant effect of message format on attitudes, $F_{1,217}$ = 5.747, *p* = 0.017, η2 = 0.026, such that the didactic message evoked more positive attitudes toward the HPV vaccination than the narrative message. Thus, H1 was supported. Although the message format approached marginal significance, it did not have any significant effect on intentions, both when the vaccine was offered for free, $F_{1,217}$ = 2.783, *p* = 0.097, η2 = 0.013 and when respondents needed to pay for it, $F_{1,217}$ = 2.264, *p* = 0.134, η2 = 0.010. Thus, H2 was rejected. Message format had a significant effect on anticipated inaction regret, $F_{1,217}$ = 5.260, *p* = 0.023, η2 = 0.024, such that the didactic message evoked greater anticipated inaction regret than the narrative message. H3 was therefore supported. Regarding anticipated action regret, message format did not show a significant effect: $F_{1,217}$ = 2.868, *p* = 0.092, η2 = 0.013. Thus, H4 was rejected. 

H5 and H6 examined the indirect effects of message format on attitudes and intentions through anticipated inaction regrets. To test these hypotheses, Preacher and Hayes’s PROCESS [44] with 5000 bootstrap samples was employed. The results showed that message form had a significant indirect effect on attitudes (*Effect* = −0.081, *p* = 0.039, 95% CI = [−0.195, −0.017]), intentions regarding the free vaccine (*Effect* = −0.158, *p* = 0.036, 95% CI = [−0.356, −0.028]), and intentions regarding the paid vaccine (*Effect* = −0.168, *p* = 0.033, 95% CI = [−0.358, −0.035]) only via anticipated inaction regret. Specifically, non-narratives increased anticipated inaction regrets (β = −0.410, *p* = 0.023), which in turn significantly increased attitudes (β = 0.197, *p* = 0.000), intentions regarding free shots (β = 0.386, *p* = 0.000), and intentions regarding paid shots (β = 0.352, *p* = 0.000). Thus, H5 and H6 were supported.

**Figure 1:**
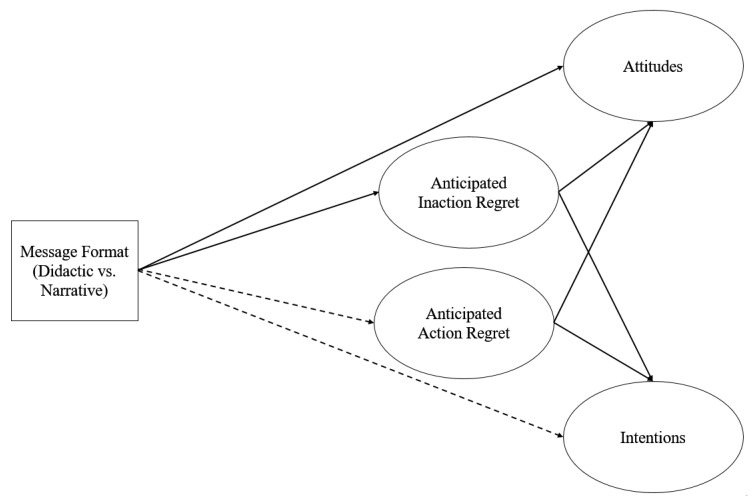
Hypotheses testing results. Solid lines indicate significant effects between variables and dotted lines indicate insignificant effects between variables.

H7 and H8 examined the indirect effects of message format on attitudes and intentions through anticipated action regrets. The same procedure using PROCESS [44] was employed. The indirect effect via anticipated action regret was not significant, although action regret significantly predicted attitudes and intentions (*p* = 0.000). Figure 1 presents a summary of the test results. Additional analyses showed that age predicted anticipated inaction regrets ($F_{1,217}$ = 9.584, *p* = 0.002), attitudes ($F_{1,217}$ = 4.560, *p* = 0.034), and intentions regarding the free shot ($F_{1,217}$ = 3.941, *p* = 0.048). Gender did not predict any of these variables.

## 4. Discussion

This is one of the first studies to focus on anticipated regrets as potential mediators in explaining narrative effects on HPV vaccination. Compared to narratives, non-narratives generally produced more positive attitudes toward HPV vaccination. Moreover, this effect occurred through anticipated inaction regret. The findings regarding message format support prior research [22] showing the relative advantage of non-narratives over narratives in HPV promotion contexts. The other two studies [1,24] found no significant differences between non-narratives and narratives when peers appeared as narrators. These mixed findings may be explained by differences in stimuli. Using a video clip, Hopfer [1] found that narratives were more effective according to a meta-analysis [21], whereas Nan et al. [24] compared narratives to statistical information rather than to the didactic information used in the present study and in [22]. One possible explanation for these mixed results is that in the context of the HPV vaccination, didactic messages could be more effective than narratives (as supported by the current study), while Hopfer’s [1] video communication mode intensified the narrative effects in her study, showing no differences across conditions. Statistical information, on the other hand, may not be as effective as didactic information in the context of HPV vaccination. To clarify, however, further research must be conducted to directly compare different types of message formats in the context of HPV vaccination. Moving beyond studies specifically examining HPV vaccination, narratives have generally been found to be more effective in other health contexts as well [21]. Taking an initial step to investigate the cause of these different results, the current study examined narratives’ indirect effects on HPV vaccinating behavior via anticipated regrets.

The present study showed that message format has indirect effects on attitudes and intentions only via anticipated inaction regret. In particular, message format did not yield a direct effect on intentions, but it did have an indirect effect on intentions via anticipated inaction regret. Prior studies have shown that inaction regret is stronger than action regret in vaccination contexts [11], since preventing cancer far outweighs any barriers to getting the vaccine such as vaccination side effects or sexual disinhibition [31]. Notably, however, both inaction and action regrets were significantly associated with attitudes and intentions but only inaction regret was significantly predicted by message format, highlighting the importance of message format.

The associations between message format and anticipated regret may provide the key to explaining narratives’ mixed effects. Generally, narratives have been found to be more effective than non-narratives. However, in HPV vaccination contexts, the effectiveness of narratives has been proven to be null and/or non-narratives have been found to be more effective, as confirmed in this study. Anticipated regrets arise in response to lost opportunities that may have improved particular situations. The choice of forgoing the HPV vaccine, compared to other health behaviors (e.g., healthy eating, exercising), is expected to incur higher levels of self-blame since it eliminates the opportunity to prevent cervical cancer by 70%. Forgoing other health behaviors may not be perceived as resulting in as much loss as forgoing the HPV vaccination. Simply put, individuals may anticipate more regret from performing or not performing behaviors that are widely perceived to protect health rather than behaviors have uncertain health outcomes.

For behaviors involving predetermined consequences, non-narratives delivered directly by medical authorities may be more effective because defying medical authorities’ recommendations can lead to significant losses in health protection. Moreover, in these cases, individuals become more vulnerable to self-blame for not complying with the recommended behaviors. For behaviors involving less predetermined consequences (e.g., exercising), individuals may doubt didactic information since the potential lost opportunities are less concrete. Therefore, individuals may become more receptive to other laypersons’ narratives, boosting narratives’ relative efficacy. Specifically, in this study, narratives provided by peers may have induced fewer anticipated regrets by being perceived as less credible, while non-narratives may have increased individuals’ expected self-blame via the perception that they were presented directly by medical experts. Although the reasons underlying mixed narrative effects cannot be fully explained, the findings of this study suggest a potential explanation for such effects and highlight the need for further research.

### 4.1. Limitations and Future Research Directions

This study’s findings need to be interpreted with a few limitations in mind. First, the sample was comprised of college students who are not representative of young adults. This sample may have been above average in terms of young adults’ socioeconomic status. The high cost of the HPV vaccine is one of the major barriers to vaccine uptake [45]. Future research should examine different groups of young adults, and such research may yield different results. In addition, participants were asked to self-report their attitudes and intentions toward vaccination immediately after being exposed to experimental messages. Individuals are less likely to take vaccines right after reading promotional messages, but their responses may change over time. In addition, attitudes and intentions often do not necessarily lead to actual behaviors [46]. Thus, future studies should examine actual behaviors using longitudinal methods.

### 4.2. Practical Implications

Despite these limitations, the findings of this study have several implications for HPV vaccine promotion message design. First, the results indicate that non-narratives are more effective than narratives in promoting HPV vaccination when the messages are presented in print form. They also show that narrative effects are mixed in this context, so health communicators should use narratives cautiously. Second, anticipated inaction regret—through which non-narratives exert their effects—was identified as an important determinant of vaccination behaviors. Health communicators could highlight the lost opportunities for cancer prevention in developing their promotional messages. In particular, the current study revealed that non-narratives affect intentions only via anticipated inaction regret. Therefore, health communicators need to strategically design messages to maximize anticipated inaction regrets. Lastly, this study’s findings regarding message format and anticipated regret can be generalized to other vaccination contexts, although further research would be required. The degree of other vaccines’ disease preventability may differ, but presenting specific information about lost opportunities for health protection by forgoing a vaccine may help enhance the effectiveness of health messages.

## 5. Conclusions

This study, by examining the relative effectiveness of narrative (versus didactic) messages on attitudes and intentions toward HPV vaccination, enhances our understanding of message format effects. As the first study to focus on anticipated inaction and action regrets, through which message format exerts its effects on vaccine uptake, this study advances scholarly knowledge of the underlying psychological processes of narrative effects. The findings provide practical implications for designing strategic messages for HPV vaccination.

## Figures and Tables

**Table 1:** Means, standard deviations, and reliabilities for key variables.

Key Variables	Didactic (*n* = 105)		Narrative (*n* = 116)		Total (*n* = 221)		
	*M*	*SD*	*M*	*SD*	*M*	*SD*	α
anticipated inaction regret	4.743 ^a^	1.314	4.379 ^b^	1.365	4.552	1.350	0.864
anticipated action regret	2.470	1.394	2.762	1.333	2.623	1.367	0.856
attitudes	5.784 ^a^	0.856	5.499 ^b^	1.044	5.634	0.968	0.946
intentions (free shot)	5.038	1.616	4.681	1.661	4.850	1.646	0.867
intentions (paid shot)	3.194	1.358	2.932	1.371	3.056	1.368	0.819

Different subscripts in a row indicate significant difference between mean scores at *p* < 0.05. α indicates the reliability for scale.

## Appendix A

Stimuli

**Condition**	**Message**
Didactic	Genital human papillomavirus (HPV) is one of the most common sexually transmitted viruses. More than half of sexually active men and women are infected with HPV at some point in their lives. HPV is usually spread through sexual contact. A survey of Korean female college students found that about 15.2% were infected with HPV. Clinical trials have shown that the HPV vaccine is highly effective in combating the high-risk HPV types responsible for cervical cancer in females and anal cancer in both males and females. The HPV vaccine effectively protects your body. Imagine the huge sense of relief you will feel after you have received the HPV vaccine!
Narrative	Genital human papillomavirus (HPV) is one of the most common sexually transmitted viruses. More than half of sexually active men and women are infected with HPV at some point in their lives. HPV is usually spread through sexual contact. A survey of Korean female college students found that about 15.2% were infected with HPV. Clinical trials have shown that the HPV vaccine is highly effective in combating the high-risk HPV types responsible for cervical cancer in females and anal cancer in both males and females. “I know the HPV vaccine works effectively to protect my body,” said Yena, a University of X student who recently received the HPV vaccination, “after I got the HPV vaccine, I felt a huge sense of relief!”

## References

[1] Hopfer S. (2012). Effects of a narrative HPV vaccination intervention aimed at reaching college women: A randomized controlled trial. Prev. Sci..

[2] World Health Organization Human Papillomavirus (HPV). http://www.who.int/immunization/diseases/hpv/en/.

[3] Garland S.M., Kjaer S.K., Muñoz N., Block S.L., Brown D.R., DiNubile M.J., Saah A.J. (2016). Impact and effectiveness of the quadrivalent human papillomavirus vaccine: A systematic review of 10 years of real-world experience. Rev. Infect. Dis..

[4] Kim J. (2018). The relationship of health beliefs with information sources and HPV vaccine acceptance among young adults in Korea. Int. J. Environ. Res. Public Health.

[5] Beavis A., Krakow M., Levinson K., Rositch A.F. (2018). Reasons for lack of HPV vaccine initiation in NIS-teen over time: Shifting the focus from gender and sexuality to necessity and safety. J. Adolesc. Health.

[6] Friedman A.L., Shepeard H. (2007). Exploring the knowledge, attitudes, beliefs, and communication preferences of the general public regarding HPV: Findings from CDC focus group research and implications for practice. Health Educ. Behav..

[7] Vamos C.A., McDermott R.J., Daley E.M. (2008). The HPV vaccine: Framing the arguments for and against mandatory vaccination of all middle school girls. J. Sch. Health.

[8] Dixon G.N., Clarke C.E. (2013). Heightening uncertainty around certain science: Media coverage, false balance, and the autism-vaccine controversy. Sci. Commun..

[9] Walter N., Ball-Rokeach S.J., Xu Y., Broad G.M. (2018). Communication ecologies: Analyzing adoption of false beliefs in an information-rich environment. Sci. Commun..

[10] Hinyard L.J., Kreuter M.W. (2007). Using narrative communication as a tool for health behavior change: A conceptual, theoretical, and empirical overview. Health Educ. Behav..

[11] Brewer N.T., DeFrank J.T., Gilkey M.B. (2016). Anticipated regret and health behavior: A meta-analysis. Health Psychol..

[12] Winterbottom A., Bekker H.L., Conner M., Mooney A. (2008). Does narrative information bias individual’s decision making? A systematic review. Soc. Sci. Med..

[13] Bruni L., Diaz M., Barrionuevo-Rosas L., Herrero R., Bray F., Bosch F.X., de Sanjosé S., Castellsagué X. (2016). Global estimates of human papillomavirus vaccination coverage by region and income level: A pooled analysis. Lancet Glob. Health.

[14] Dunne E.F., Unger E.R., Sternberg M., McQuillan G., Swan D.C., Patel S.S., Markowitz L.E. (2007). Prevalence of HPV infection among females in the United States. J. Am. Med Assoc..

[15] Kreuter M.W., Green M.C., Cappella J.N., Slater M.D., Wise M.E., Storey D., Clark E.M., O’Keefe D.J., Erwin D.O., Holmes K. (2007). Narrative communication in cancer prevention and control: A framework to guide research and application. Ann. Behav. Med..

[16] Janssen E., van Osch L., de Vries H., Lechner L. (2013). The influence of narrative risk communication on feelings of cancer risk. Br. J. Health Psychol..

[17] Yoo J.H., Kreuter M.W., Lai C., Fu Q. (2014). Understanding narrative effects: The role of discrete negative emotions on message processing and attitudes among low-income African American women. Health Commun..

[18] Green M.C. (2006). Narratives and cancer communication. J. Commun..

[19] Escalas J.E. (2004). Imagine yourself in the product: Mental simulation, narrative transportation, and persuasion. J. Advert..

[20] Green M.C., Brock T.C. (2000). The role of transportation in the persuasiveness of public narratives. J. Personal. Soc. Psychol..

[21] Shen F., Sheer V.C., Li R. (2015). Impact of narratives on persuasion in health communication: A meta-analysis. J. Advert..

[22] Kim J., Nan X. (2019). Temporal framing effects differ for narrative versus non-narrative messages: The case of promoting HPV vaccination. Commun. Res..

[23] Nan X., Futerfas M., Ma Z. (2017). Role of narrative perspective and modality in the persuasiveness of public service advertisements promoting HPV vaccination. Health Commun..

[24] Nan X., Dahlstrom M.F., Richards A., Rangarajan S. (2015). Influence of evidence type and narrative type on HPV risk perception and intention to obtain the HPV vaccine. Health Commun..

[25] Zeelenberg M., Pieters R. (2007). A theory of regret regulation 1.0. J. Consum. Psychol..

[26] Smerecnik C.M., Ruiter R.A. (2010). Fear appeals in HIV prevention: The role of anticipated regret. Psychol. Health Med..

[27] van Empelen P., Kok G., Jansen M., Hoebe C. (2001). The additional value of anticipated regret and psychopathology in explaining intended condom use among drug users. Aids Care.

[28] Abraham C., Sheeran P. (2004). Deciding to exercise: The role of anticipated regret. Br. J. Health Psychol..

[29] Conner M., Sandberg T., McMillan B., Higgins A. (2006). Role of anticipated regret, intentions and intention stability in adolescent smoking initiation. Br. J. Health Psychol..

[30] Connolly T., Reb J. (2003). Omission bias in vaccination decisions: Where’s the “omission”? Where’s the “bias”?. Organ. Behav. Hum. Decis. Process..

[31] Ziarnowski K.L., Brewer N.T., Weber B. (2009). Present choices, future outcomes: Anticipated regret and HPV vaccination. Prev. Med. Int. J. Devoted Pract. Theory.

[32] Beike D.R., Markman K.D., Karadogan F. (2009). What we regret most are lost opportunities: A theory of regret intensity. Personal. Soc. Psychol. Bull..

[33] Roese N.J., Summerville A. (2005). What we regret most … and why. Personal. Soc. Psychol. Bull..

[34] Ferguson E., Gallagher L. (2007). Message framing with respect to decisions about vaccination: The roles of frame valence, frame method and perceived risk. Br. J. Psychol..

[35] Chapman G.B., Coups E.J. (2006). Emotions and preventive health behavior: Worry, regret, and influenza vaccination. Health Psychol..

[36] Cox D., Sturm L., Cox A.D. (2014). Effectiveness of asking anticipated regret in increasing HPV vaccination intention in mothers. Health Psychol..

[37] McQueen A., Kreuter M.W., Kalesan B., Alcaraz K.I. (2011). Understanding narrative effects: The impact of breast cancer survivor stories on message processing, attitudes, and beliefs among African American women. Health Psychol..

[38] Faul F., Erdfelder E., Lang A.G., Buchner A. (2007). G* Power 3: A flexible statistical power analysis program for the social, behavioral, and biomedical sciences. Behav. Res. Methods.

[39] Braddock K., Dillard J.P. (2016). Meta-analytic evidence for the persuasive effect of narratives on beliefs, attitudes, intentions, and behaviors. Commun. Monogr..

[40] Korea Centers for Disease Control and Prevention Human Papillomavirus (HPV). https://nip.cdc.go.kr/irgd/index.html.

[41] Orbell S., Hagger M. (2006). Temporal framing and the decision to take part in Type II Diabetes screening: Effects of individual difference in consideration of future consequences. Health Psychol..

[42] Kim J., Nan X. (2016). Effects of consideration of future consequences and temporal framing on acceptance of the HPV vaccine among young adults. Health Commun..

[43] Nan X. (2012). Communicating to young adults about HPV vaccination: Consideration of message framing, motivation, and gender. Health Commun..

[44] Preacher K.J., Hayes A.F. (2004). SPSS and SAS procedures for estimating indirect effects in simple mediation models. Behav. Res. Methods Instrum. Comput..

[45] Brewer N.T., Fazekas K.I. (2007). Predictors of HPV vaccine acceptability: A theory-informed, systematic review. Prev. Med..

[46] Ajzen I., Czasch C., Flood M.G. (2009). From intentions to behavior: Implementation intention, commitment, and conscientiousness. J. Appl. Soc. Psychol..

